# Supplementary figures and images for: Autism-related KLHL17 and SYNPO act in concert to control activity-dependent dendritic spine enlargement and the spine apparatus
Source: PLoS Biol. 2023 Aug 31;21(8):e3002274. doi: 10.1371/journal.pbio.3002274 (PMC10499226; doi:10.1371/journal.pbio.3002274)

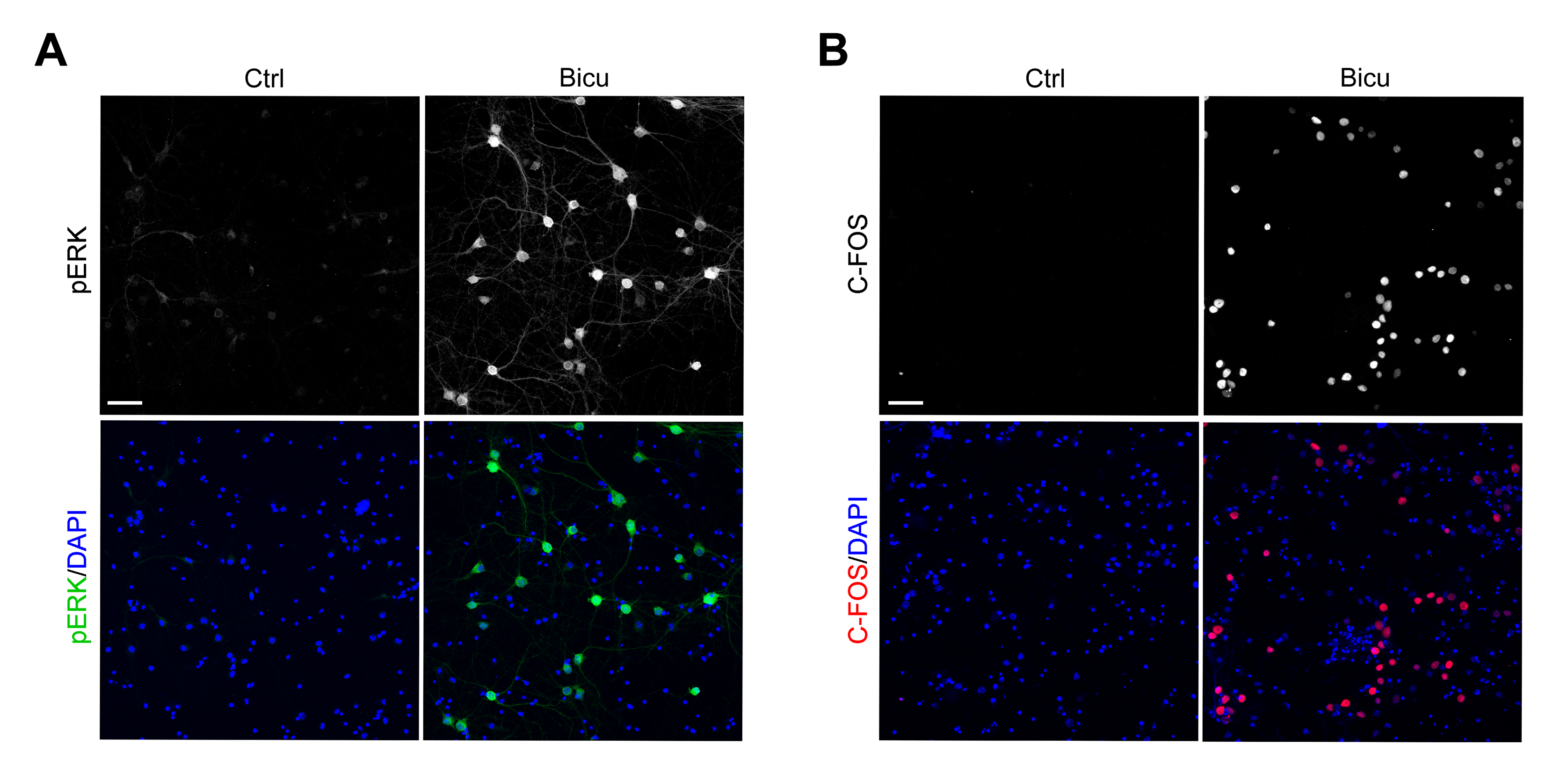

Supplement: S1 Fig — Wild-type neurons at 18 DIV were treated with bicuculline (40 μM) for 15 min to investigate ERK phosphorylation (A) or for 2 h to assess expression of C-FOS (B). The levels of ERK phosphorylation and C-FOS expression are very low in the absence of bicuculline under our experimental conditions. Scale bars: (A), (B) 50 μm. (TIF) [file pbio.3002274.s001.tif]

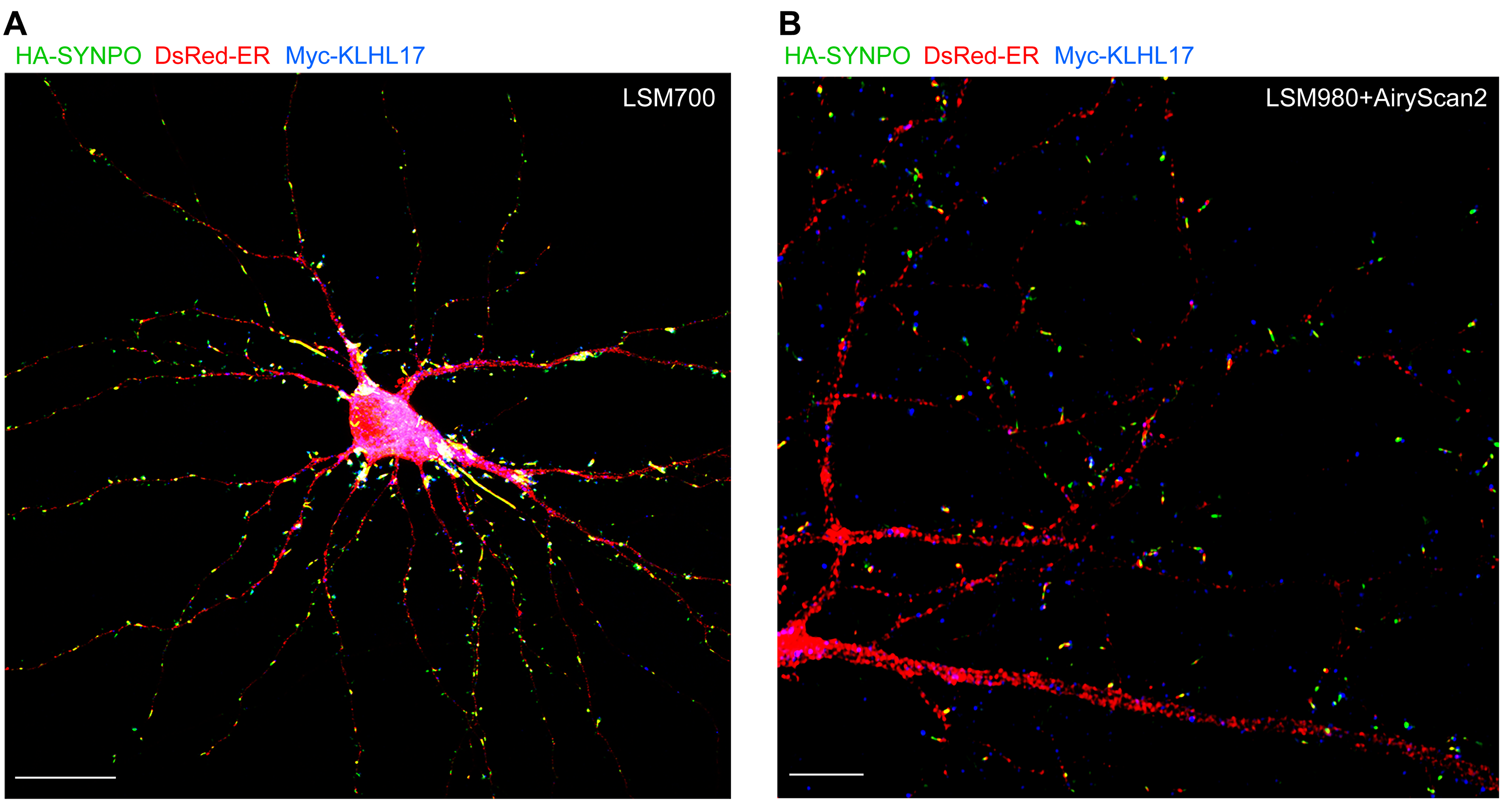

Supplement: S2 Fig — (A) Enlarged image of the left panel in Fig 6A, which was acquired using a LSM700 system. (B) Enlarged image of the left panel in Fig 6C, which was acquired using an LSM980 system with AiryScan2. Scale bar: (A) 20 μm; (B) 5 μm. (TIF) [file pbio.3002274.s002.tif]

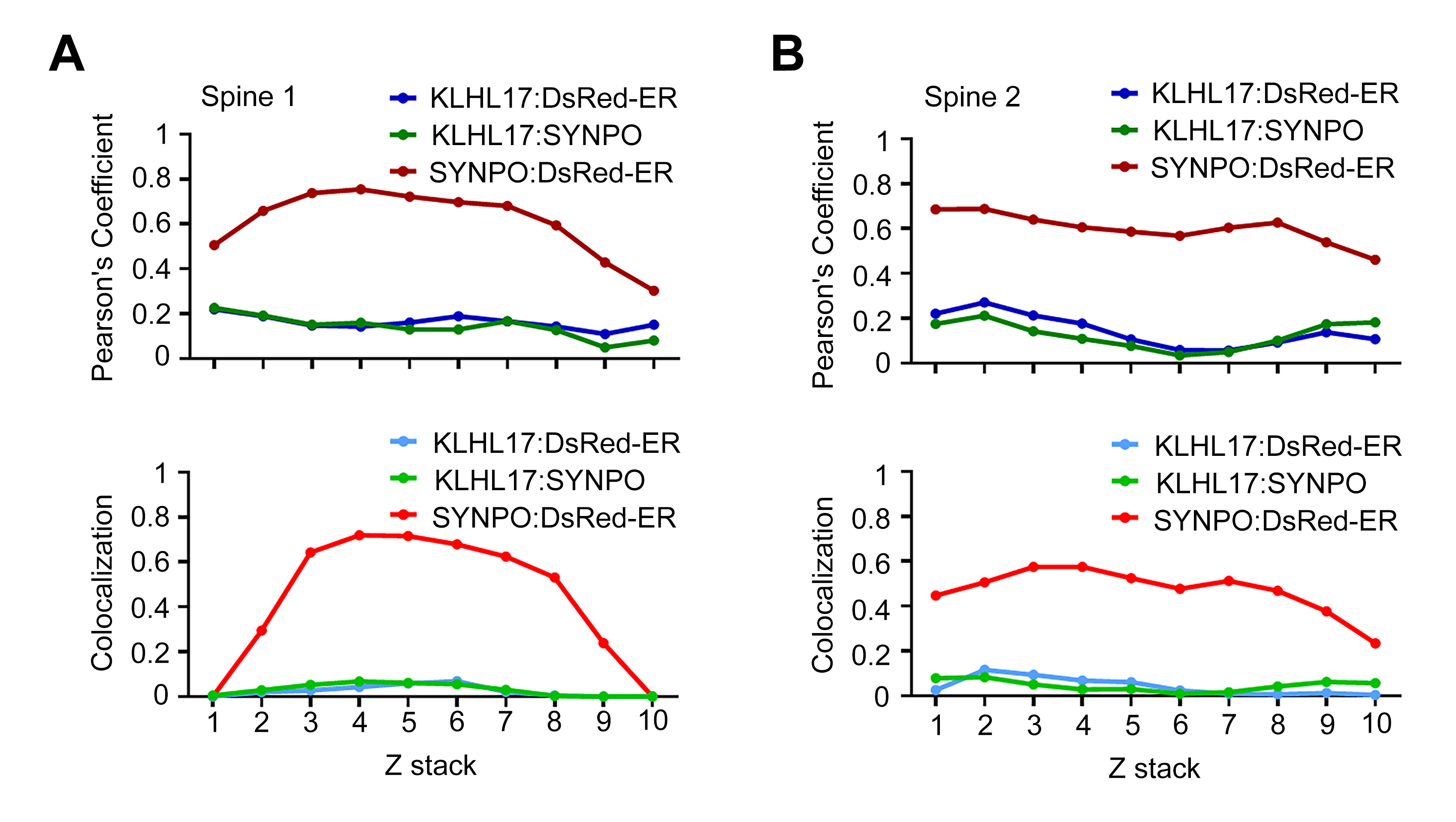

Supplement: S3 Fig — The correlation and colocalization of KLHL17, SYNPO, and ER in the spine apparatuses shown in Fig 10C were analyzed using ImageJ/FIJI. (A) Spine 1. (B) Spine 2. Upper panel, Pearson’s correlation; lower panel, colocalization percentage. X-axis: section number of Z-series. The numerical value data are available in S1 Data. (TIF) [file pbio.3002274.s003.tif]

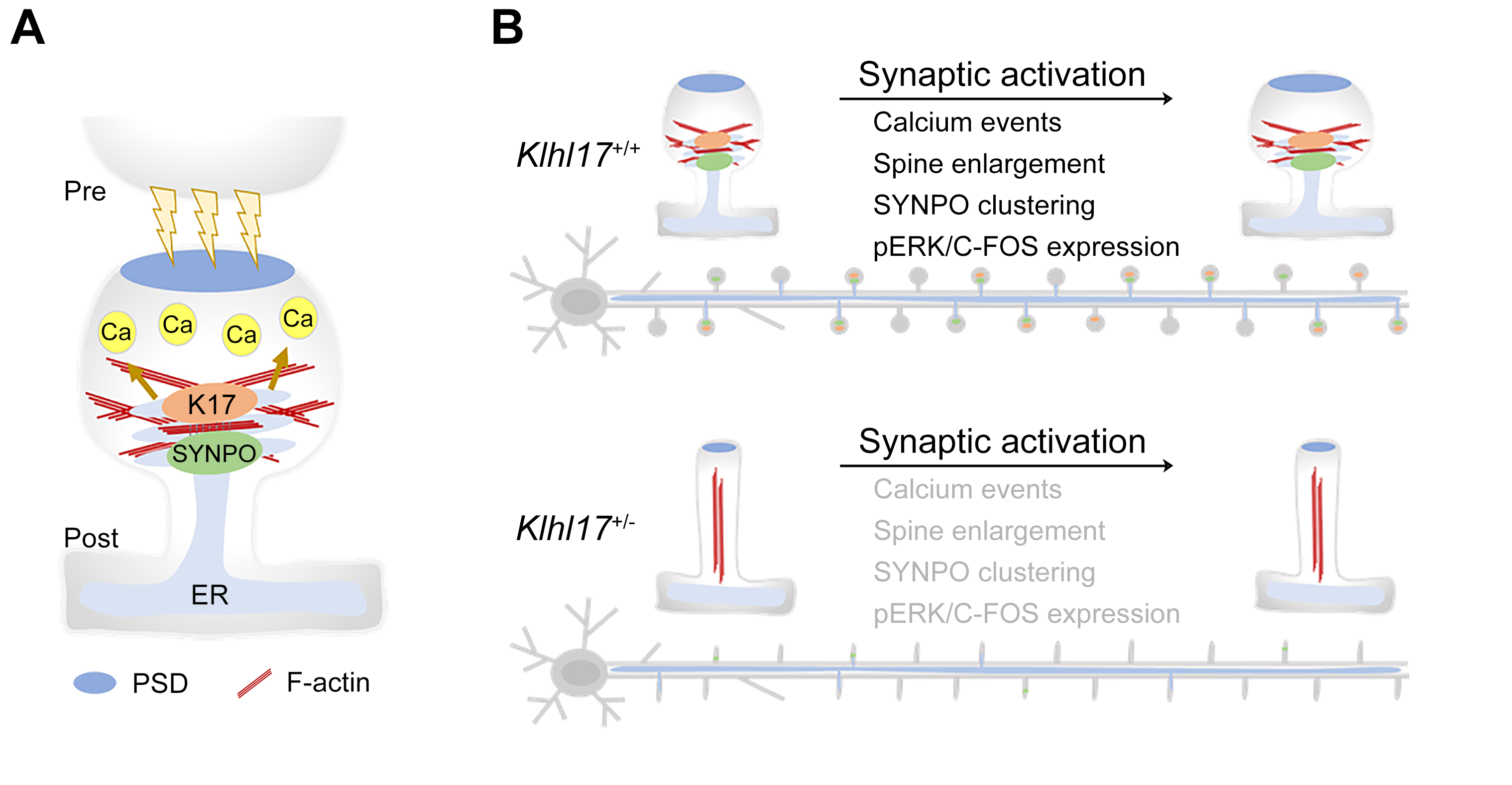

Supplement: S4 Fig — (A) KLHL17 associates with SYNPO, a marker of the spine apparatus, to control the synaptic ER distribution, thereby influencing calcium dynamics at dendritic spines and consequently regulating activity-dependent events. (B) Comparison of Klhl17-deficient mice and wild-type mice. (TIF) [file pbio.3002274.s004.tif]

Fig. 1A

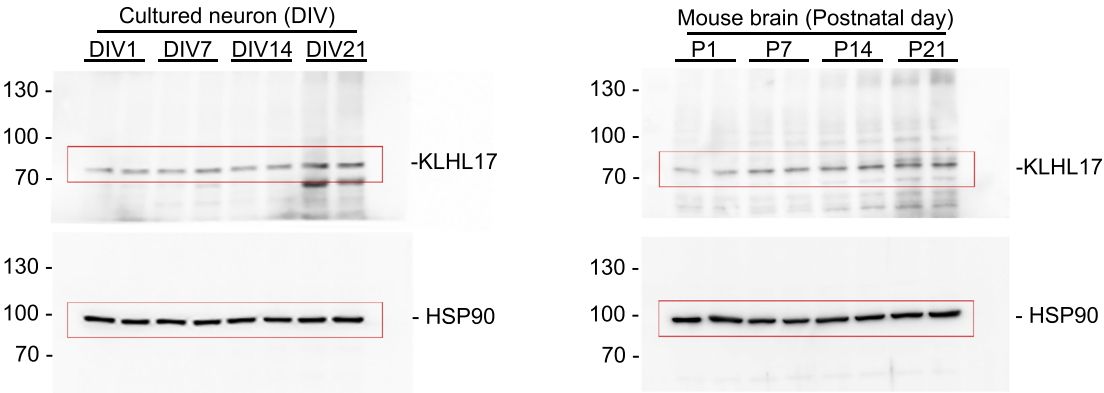

Fig. 1C

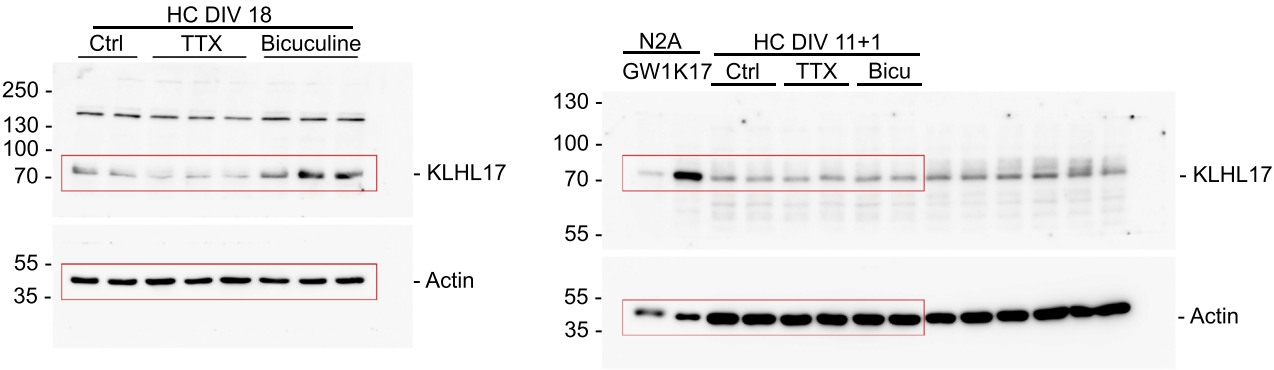

Fig. 1J

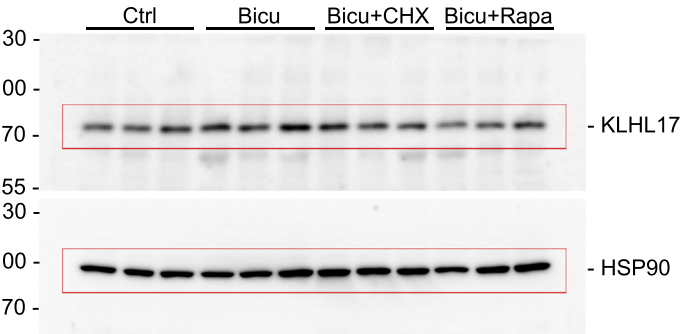

Fig. 3D

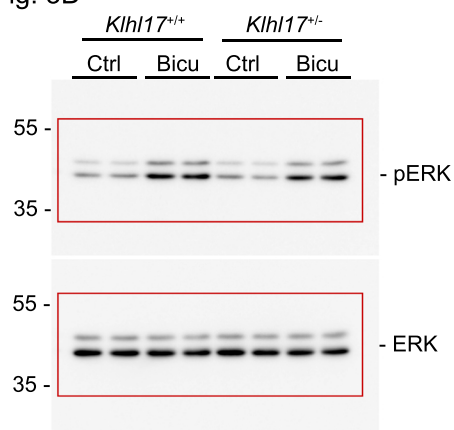

Fig. 6E

| 2% Input |     | IP:Myc |     |
|----------|-----|--------|-----|
| +SYNPO   |     | +SYNPO |     |
| Ctrl     | K17 | Ctrl   | K17 |

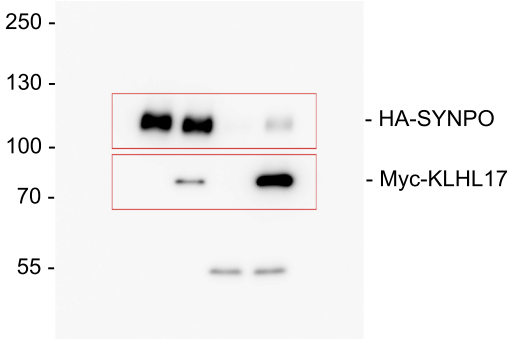

Fig. 8B

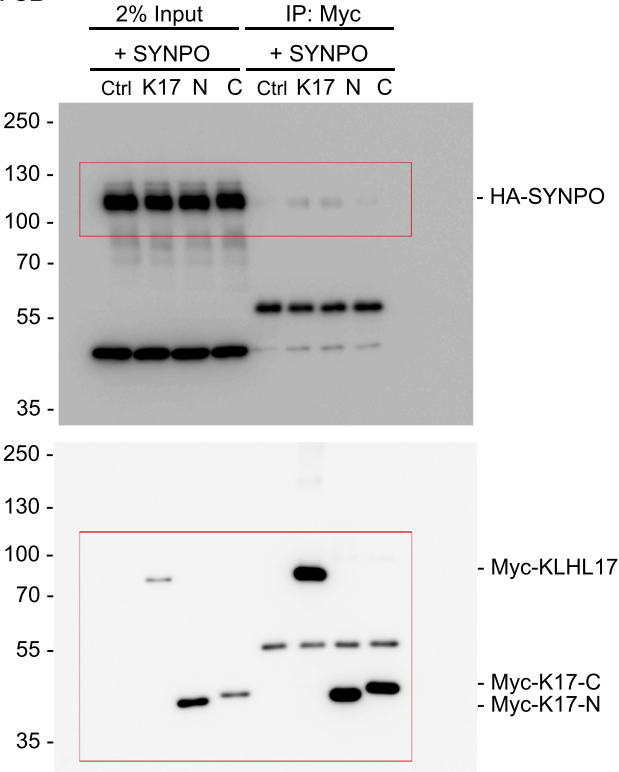

Fig. 8C

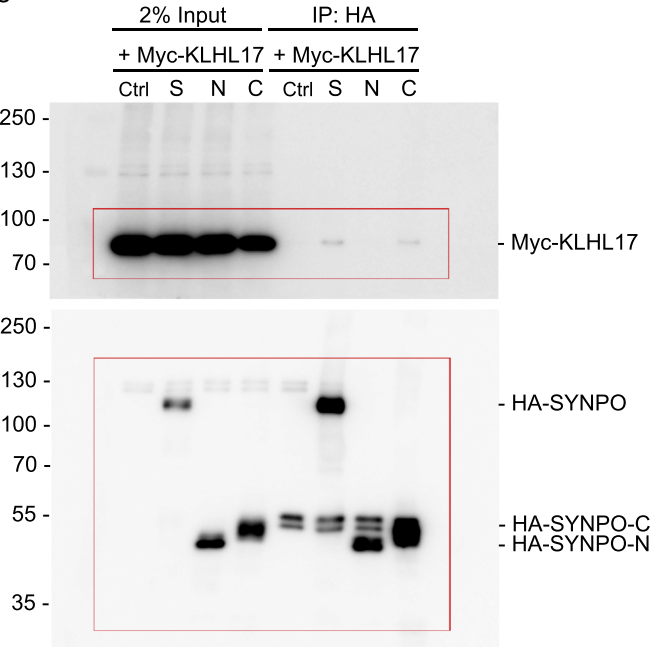

Supplement: S1 Raw Images — (PDF) [file pbio.3002274.s005.pdf]
