# Supplementary material for: Autism-related KLHL17 and SYNPO act in concert to control activity-dependent dendritic spine enlargement and the spine apparatus
Source: PLoS Biol. 2023 Aug 31;21(8):e3002274. doi: 10.1371/journal.pbio.3002274 (PMC10499226; doi:10.1371/journal.pbio.3002274)
Supplement: Attachment — Submitted filename: Rebuttal_20230522.pdf [file pbio.3002274.s008.pdf]

Our Response to Reviewers

Reviewer #1:

Reviewer #1's general comments: *Hu et al. study the importance of two postsynaptic proteins ,KLHL17 (actinfilin) and synaptopodin, on the structure and function of dendritic spines. They find that KLHL17 is upregulated during development by neuronal activity (NMDAR-dependent). While in wild-type neurons high activity leads to enlarged spine heads, this is not the case in KLHL17 knock-down or knock-out neurons. KLHL17 deficiency reduces ERK and FOS expression after high activity and reduces the frequency of spontaneous calcium events in spines. Fewer spines contain endoplasmic reticulum. As they show with conventional confocal microscopy and Airyscan, KLHL17 colocalizes tightly with synaptopodin in dendritic spines, suggesting it is part of the spine apparatus. Indeed, KLHL17 deficiency or KO reduces the fraction of spines containing a spine apparatus, and synaptopodin overexpression rescues the deficits of KLHL17-deficient neurons. Overexpression of truncated forms of synaptopodin suggests that binding of KLHL17 to synaptopodin (perhaps indirect) is needed for efficient spine apparatus formation and spine head enlargement.*

The study is easy to read and understand due to its clear structure and logic. The data quality is excellent, statistical tests are appropriate and all controls are in place. The role of KLHL17 in spine apparatus assembly and spine head structure is clearly demonstrated, providing a possible mechanistic explanation for its synaptic function and association with autism. I have only minor suggestions for improvements.

Our response to Reviewer #1's general comments:

We very much appreciate Reviewer #1's positive comments and strong support.

Reviewer #1's minor point #1*: Figure 4B and E: Please provide relative change in fluorescence (dF/F) instead of relative fluorescence units (RFU) as a measure of calcium concentration changes. Although spine calcium transients were analyzed in spine heads, the example (A) shows a dendritic calcium wave. Could you provide a more detailed analysis of dendritic vs. spine head Ca signals? For example, showing in (B) the fluorescence time course in both spine head and parent dendrite. Ideally, it would be nice to analyze the amplitude of spine Ca transients that are NOT associated with dendritic Ca events. Right now, the differences could be explained by lower levels of spontaneous activity in the +/- cultures. The open question is: Does KLHL17 deficiency (=lack of spine ER) directly affect spine calcium handling? Perhaps a discussion point: The higher Ca amplitudes could be due to the smaller volume of spines in KLHL17+/- neurons (less dilution).*

Our response to Reviewer #1's minor point #1:

In fact, we presented dF/F data in our original manuscript but erroneously labeled them as relative fluorescence units (RFU). We apologize for this mistake, which has now been corrected in the revised manuscript. We very much thank Reviewer #1 for this point.

In addition to total calcium events at spines shown in the original manuscript, we have reanalyzed the results of calcium imaging by defining paired ROIs, one of which was located at the spine head and the other was placed in the dendrite close to a location where dendritic spines emerged (see **new Figure 4F** as an example). By analyzing the paired ROIs at the spine and dendrite, we could determine spine-only, dendrite-only and paired (both spine and dendrite) events. Under our experimental condition, the frequency of spine- or dendrite-only calcium events was very low for both Klhl17+/– and wild-type neurons. However, for paired events, Klhl17+/– neurons exhibited significantly fewer calcium events compared to wild-type neurons. Moreover, we measured the amplitude of the calcium events. Since we were interested in the calcium events at spines, the amplitude at the spine of the paired events was compared to spine-only and dendrite-only events. We found that the amplitudes of spine-only and paired

calcium events were higher in Klhl17+/– neurons relative to wild-type neurons. However, the amplitudes of dendrite-specific events were indistinguishable between Klhl17+/– and wild-type neurons. Thus, the reduced frequency of total calcium events at dendritic spines is attributable to paired events, whereas both spine-only and paired events account for the enhanced amplitude in Klhl17+/– neurons. These new results also imply that the localized alteration of ER organization elicited by Klhl17 deficiency likely influences calcium dynamics at dendritic spines. We have included these new data as **new Figure 4F-4I** in the revised manuscript.

Finally, regarding whether or not higher calcium amplitude is due to a smaller spine volume in KLHL17+/- neurons, although we do not have direct evidence, we think it is less likely that the enhanced amplitude is caused by lower dilution because of our results on SYNPO-C. In our study, we found that SYNPO-C expression reduced both the amplitude of calcium events (**Figure 9D**) and the width of dendritic spine heads (**Figure 9E, 9G**). Thus, smaller spine heads are not always correlated with a higher amplitude of calcium events. Our data also suggest that the enhanced amplitude of calcium events caused by Klhl17 deficiency may not be relevant to SYNPO, with another molecule or mechanism perhaps being involved. Nevertheless, we have strengthened this point in the Discussion section of the revised manuscript.

Reviewer #1's minor point #2*: Fig. 5D: SPH+ ("spine head-positive") is a very unusual term and abbreviation, I had to search to find an explanation in the text. Would this correspond to the frequently used categories 'mushroom' and 'thin'? Please find a better label for the columns, even if it needs a bit more space.*

Our response to Reviewer #1's minor points #2:

Acknowledged. We have changed the terms accordingly. We thank Reviewer #1 for this suggestion.

Reviewer #1's minor point #3*: Page 12: "cytoskelens"*

Our response to Reviewer #1's minor points #3:

Apologies. We have corrected this error. Thank you for pointing it out.

Reviewer #1's minor point #4*: Title: Too long, the first half is sufficient.*

Our response to Reviewer #1's minor points #4:

We have now shortened the title of the revised manuscript to "**Autism-related KLHL17 and SYNPO act in concert to control activity-dependent dendritic spine enlargement and the spine apparatus**". Again, we thank Reviewer #1 for her/his strong support, comments and corrections.

Reviewer #2:

Reviewer #2's general comments: *This work from Hu and colleagues explores the roles of KLHL17, a protein which mutation is associated with autism spectrum disorder. The authors identify a possible mechanism for the regulation of activity-dependent dendritic spines enlargement mediated by KLHL17. They suggest that the associated action of KLHL17 and synaptopodin promotes the insertion of the spine apparatus (ER) in dendritic spines and this effect is responsible for the dendritic spine enlargement, possibly thanks to the Calcium released by the ER in this compartment.*

The experiments are of excellent quality and the story is of great interest for the cellular neuroscience field.

Our response to Reviewer #2's general comments:

We very much appreciate Reviewer #2's positive comments and strong support.

Reviewer #2's major point #1*: I feel like the evidence that KLHL17 regulates dendritic spine enlargement via SYNPO and via the insertion of the spine apparatus in dendritic spines is somehow still*

unclear, especially considering the importance of actin polymerisation in this phenomenon and the already demonstrated role of KLHL17 on actin polymerisation. It is also possible that KLHL17 regulates actin polymerization, causing smaller dendritic spines which are not able to accommodate ER, without a direct action of KLHL17 on the ER. One experiment would be required:
- does reduction of SYNPO levels in a KLHL17 -/- background further affect spine apparatus insertion and activity dependent spine enlargement? This would clarify if they participate together in this process or they have independent actions on the same process.

Our response to Reviewer #2's major point #1:
Given that SYNPO-C is involved in the association between KLHL17 and SYNPO (though likely through an indirect mechanism) and that, similar to *Klhl17* deficiency, SYNPO-C expression, but not SYNPO-N, results in an impaired dendritic spine distribution of ER, rather than reducing SYNPO levels, we used SYNPO-C overexpression in Klhl17–/– neurons to demonstrate that KLHL17 and SYNPO act together to control ER distribution. We anticipated this experimental approach to be more specific than SYNPO knockdown because SYNPO knockdown may influence other SYNPO functions. Moreover, the results using SYNPO-C are consistent with other data using SYNPO-C in the original manuscript.

Similar to the data shown in our original manuscript (**Figure 8D-8F**), our new set of results revealed that fewer than 15% of the dendritic spines of Klhl17–/– neurons contained ER (**new Figure 8G-8I**). In contrast to wild-type neurons, SYNPO-C overexpression in Klhl17–/– neurons did not further reduce the percentage of ER-positive spines. The results are similar to the levels of neurons expressing SYNPO-N and transfected with vector control. Since SYNPO-C expression did not further impair the spine distribution of ER, this outcome suggests that KLHL17 and SYNPO indeed work together to control ER distribution into dendritic spines. These new data have been included as **new Figure 8G-8I** in the revised manuscript.

Reviewer #2's major point #2: Figure 4: that the changes in frequency and amplitude of calcium events upon KLHL17 reduction are dependent on a reduced efflux of calcium from the ER is not demonstrated. The same experiment should be performed with NMDA-R blockers (is the defect still visible) and separately with blockers of ER calcium channels.

Our response to Reviewer #2's major point #2:
In **Figure 1H** of our original manuscript, it is clear that the NMDAR pathway regulates protein levels of KLHL17 in mature neurons. Therefore, it would not be appropriate to disrupt NMDAR signaling for our purposes. Nevertheless, given that demonstrating an involvement of ER in KLHL17-regulated calcium dynamics is more relevant, instead we have conducted further experiments in which we added ryanodine to block the ryanodine receptor, a calcium channel localized at ER, in both WT and Klhl17–/– neurons. We found that ryanodine treatment reduced the frequency, but not amplitude, of calcium events in wild-type neurons. Importantly, neither the frequency nor amplitude of calcium events in Klhl17–/– neurons was affected by ryanodine treatment. These new results are consistent with our original finding that synaptic ER distribution is greatly reduced by Klhl17 deficiency. The new data from our ryanodine experiment have been added to the revised manuscript as **new Figure 7A-7D**.

Reviewer #2's major point #3: Can the author perform calcium imaging experiments with and without KLHL17 upon the activity paradigm implemented in all other experiments (15 min Bicuculline + recovery)?

Our response to Reviewer #2's major point #3:
Initially, we did try to measure calcium levels after bicuculline treatment. However, the neurons responded strongly to bicuculline treatment, resulting in a long-term plateau of calcium signals. These saturated signals were maintained for almost the entire recording period. Perhaps the change in calcium concentration induced by bicuculline was much greater than the detection range of GCaMP6s, *i.e.*, the calcium sensor used in our study. Consequently, we have not conducted calcium imaging after

bicuculline treatment. Instead, to measure long-term effects, we measured ERK phosphorylation and C-FOS expression.

***Reviewer #2's major point** #4: The ER or near ER localisation of KLHL17 is somehow not fully convincing. The airyscan2 imaging used in Figure 6C is not a full super-resolution approach with a nominal resolution of 90 nm (and realistically the resolution will be lower). Would it be possible for the authors to perform STORM or STED experiments for this? Also imaging the endogenous KLHL17 and SYNPO would be important.*

Our response to Reviewer #2's major point #4:

Unfortunately, neither STORM nor STED is available to us. Therefore, we could not perform the suggested analysis. Nevertheless, fortunately, Dr. Bi-Chang Chen, one of our collaborators, recently modified the expansion microscopy method by using trypsin to replace proteinase K, which enables retention of more protein fragments and fluorescence signals in a hydrogel (Wang et al., 2023, bioRxiv 10.1101/2023.03.20.533392). Therefore, we have applied that trypsin protocol to investigate the distribution of ER, SYNPO and KLHL17 in cultured hippocampal neurons. We found that our cultured hippocampal neurons could be expanded 3.5-fold (n**ew Figure 10A**). Since the original resolution of our LSM980 system with Airyscan2 is 90~120 nm, the 3.5-fold expansion renders the resolution of our system up to 26~35 nm after expansion, thus significantly enhancing image resolution.

Our state-of-the-art expansion microscopy approach did not generate obvious morphological distortions because we obtained the entire and continuous neuronal morphology after expansion (**New Figure 10A-10B**). Moreover, the fine morphological structures of filopodia and axons were also well preserved (**New Figure 10B**). Using this system, we could examine in fine 3D detail the structure of ER, as well as SYNPO and KLHL17 distributions, in dendritic spines. Through Imaris-based processing, we found that SYNPO and ER signals were intermingled with each other and that KLHL17 was adjacent to the SYNPO/ER complex or at the front of the complex toward the dendritic spine tip (**new Figure 10C**). Some KLHL17 signals were also intermingled with the ER/SYNPO complexes (**new Figure 10C**). Our 3D images also revealed complex structures containing KLHL17, SYNPO and ER (**new Figure 10C**, right). These super-resolution images further strengthen our model that KLHL17 controls the spine apparatus.

We would certainly have loved to image the endogenous KLHL17, SYNPO and ER markers. However, since we could not find suitable antibodies derived from different species for double or triple staining of endogenous proteins, we could not conduct the suggested experiment. Previous studies have indicated that exogenous SYNPO and KLHL17 exhibit similar distribution patterns to those of endogenous proteins (Falahati et al., 2022, PNAS 119:e2203750119; Hu et al., 2020, Journal of Biomedcial Science 27:103). Therefore, we used Myc- and HA-tagged proteins and DsRed-ER in our study to investigate the distributions of these two proteins, as well as ER, in dendritic spines.

Finally, we very much appreciate Reviewer #2 for suggesting to conduct super-resolution imaging, which has significantly enhanced the impacts of our study.

***Reviewer #2's major point #5.** I do not think that showing statistical analysis per "dendritic segment" or per "spine" is acceptable. At least the analysis should be shown per neuron averaging the dendritic segments or the spines belonging to the same neurons.*

Our response to Reviewer #2's major point #5:

As a pertinent example, if we want to understand the average body weight and height of teenagers in a city, we might select perhaps 10 schools in that city and record the body weight and height of let's say 100 students in each of those schools. Thus, a total of 1000 students from 10 schools represent the subjects. Then, we average the individual body weight and height of those 1000 students to obtain an average body weight and height of all students. We would not first average the data for the 100 students in each school and then average the data of the 10 schools. Similarly, when we investigate the features

of “dendritic spines”, one spine represents one sample. When we examine “the spine density of dendrites”, one dendritic segment is one sample. We usually collect three dendritic segments from each neuron and 10-20 neurons from at least two different preparations for analysis. We wish to emphasize that our experiments are routinely performed in a blind manner because the samples are relabeled by another person in the lab before conducting imaging and analysis, thereby avoiding potential bias. We also consulted experts in the Institute of Statistical Science, Academia Sinica (https://disc.stat.sinica.edu.tw/en/about-us/), who have confirmed that our statistical analysis is appropriate. In addition, both Reviewers #1 and #3 are satisfied with our statistical analysis. Accordingly, and respectfully, we do not think it is appropriate to change it.

Reviewer #2's major point #6. *All bar graphs should show individual data points.*
Our response to Reviewer #2's major point #6:
Accepted. Individual data points are now shown in all plots of the revised manuscript.

Reviewer #2's minor point #1. *Figure 1F; GFP/Myc-KLHL17 label is unclear of what is shown in the image. I think it should say myc-KLHL17 only.*
Our response to Reviewer #2's minor point #1:
Apologies, we have now relabeled the figure to make it clear.

Reviewer #2's minor point #2. *Figure 1H; the intensity of myc-KLHL17 in the image in the panel Bicu+CHX is still higher than ctrl, and as such not representative of what is shown in the bar graph in panel I.*
Our response to Reviewer #2's minor point #2:
Acknowledged, the image we presented is not sufficiently representative when we reviewed our quantification results. Therefore, we have replaced it with another more appropriate image. We thank Reviewer #2 for his/her careful assessment.

Reviewer #2's minor point #3. *Figure 3: Can the authors please add pERK and c-Fos images at baseline condition?*
Our response to Reviewer #2's minor point #3:
Before comparing the responses of Klhl17+/– and wild-type neurons to bicuculline treatment, we did examine the signals at baseline, *i.e.*, in the absence of bicuculline treatment. We found the signals of these baseline controls to be extremely low (**New S1 Fig**), and so we felt that presenting those data for all comparisons would not be particularly meaningful. Nevertheless, we do agree to include this information, which has now been added into the revised manuscript as **new S1 Fig**. We thank Reviewer #2 for this suggestion.

Reviewer #2's minor point #4. *Figure 3: the DAPI signal is not really visible when the figure is printed, can the author increase brightness?*
Our response to Reviewer #2's minor point #4:
Apologies, we have replaced the representative images in **Figure 3** with those displaying stronger DAPI signals.

Reviewer #2's minor point #5. *Figure 3: Can the authors show pERK also via western blotting together with total ERK intensity? This is important because it would allow to show pERK/ERK signal.*
Our response to Reviewer #2's minor point #5:
Agreed. We now include the immunoblots of pERK and total ERK as **new Figure 3D-3E** in the revised manuscript. Baseline controls are included in this data.

Reviewer #2's minor point #6. *Figure 5A. Can the authors please show individual colour panels?*
Our response to Reviewer #2's minor point #6:
We did show the individual panels and merged color images of representative dendritic spines in **original Figure 5A**. Based on Reviewer #2's suggestion, we now show the individual color panels as B/W images of the whole cell image in **Figure 5A**.

Reviewer #2's minor point #7. *Figure 5E: the GFP outline is not visible when the figure is printed. Can the authors make it brighter or thicker?*
Our response to Reviewer #2's minor point #7:
Apologies, we have now made the outlines wider in **revised Figure 5E**.

Reviewer #2's minor point #8. *Figure 6F, I: the GFP outline is not visible when the figure is printed. Can the authors make it brighter or thicker?*
Our response to Reviewer #2's minor point #8:
Apologies, as for **Figure 5E**, we have now made the outlines thicker.

Reviewer #2's minor point #9. *Figure 6A and C: the image is very dim when printed, can the authors make it brighter.*
Our response to Reviewer #2's minor point #9:
We feel Reviewer #2 may perceive the images of **Figures 6A** and **6C** to be too dim when printed because the background is black and some signals are of small puncta. However, we are hesitant to make these images brighter because then some of the signals will become oversaturated. To make these images easier to view on paper, we have enlarged the images and included them in **new S2 Fig** of the revised manuscript.

Reviewer #2's minor point #10. *Fig 7I: as before, can the authors measure pERK and ERK via western blot and show pERK/ERK ratio?*
Our response to Reviewer #2's minor point #10:
We analyzed transfected neurons in **Figure 7M**. Since the transfection efficiency of cultured neurons is low (~1%), we cannot use immunoblotting to analyze altered activity. That is the reason why we originally adopted immunostaining to analyze ERK activation.

Reviewer #2's minor point #11. *Figure 8 B: coIPed HA is barely visible, can the author show a longer exposure image?*
Our response to Reviewer #2's minor point #11:
Apologies, we have now included long-exposure images of HA-SYNPO in **Figure 8B** and Myc-KLHL17 in **Figure 8C**.

Reviewer #2's minor point #12. *Figure 8D-F: it would be interesting to see what effect the expression of SYNPO full length has on the percentage of dendritic spines positive for ER?*
Our response to Reviewer #2's minor point #12:
In **Figure 8D-8F**, we aimed to analyze the effect of SYNPO-C on the synaptic distribution of ER. Since SYNPO-N does not associate with KLHL17, we used SYNPO-N as a negative control for SYNPO-C. Therefore, we did not include full-length SYNPO because it is not the focus of **Figure 8D-8F**. Consequently, the absence of full-length SYNPO does not influence our interpretations.

Reviewer #2's minor point #13. *Figure 8D-F: it would be important to measure the expression levels of Synpo-N and Synpo-C to verify they are similar.*

Our response to Reviewer #2's minor point #13:
We present the expression levels of full-length SYNPO, SYNPO-N and SYNPO-C in Neuro-2A cells in **Figure 8C** (input lanes) based on immunoblotting. Both SYNPO-N and SYNPO-C are expressed at higher levels than full-length SYNPO, with slightly higher expression of SYNPO-C relative to SYNPO-N. The results of our immunostaining clearly show that their expression patterns in neurons are distinct. Thus, it is difficult to quantify SYNPO-C and SYNPO-N expression levels in neurons. SYNPO-N signal was more diffuse and that of SYNPO-C remained punctate (**original Figure 8D, lower panel**), which likely reflects the different protein-protein interactions and functions of these two peptides. Consistently, we found that only SYNPO-C but not SYNPO-N could disrupt ER entry into dendritic spines (**original Figure 8D**).

Reviewer #2's minor point #14. *Figure 9I: as before, can the authors measure pERK and ERK via western blot and show pERK/ERK ratio?*
Our response to Reviewer #2's minor point #14:
Only transfected neurons were analyzed in **Figure 9I**. Therefore, we did not perform immunoblotting for this set of experiments.

Reviewer #3:
Reviewer #3's general comments: *Reviewer #3: In this very interesting manuscript the authors report the discovery and characterization, as well as the functional impact, of the interaction between two dendritic spine proteins, KLHL17 and SYNPO. Dendritic spine plasticity, as well as these genes, are involved in neurodevelopmental disorders such as autism, hence the work has both basic and translational relevance. The experiments are well-designed, proper controls and statistics are used, and the experiments are performed at high standards of quality. The logical flow is correct. The major findings are that KLHL17 expression is regulated by neuronal activity, and it interacts directly with synaptopodin, a protein associated with ER in dendritic spines. They then characterize this interaction, and its effects on ER in spines, neuronal activity, dendritic spine morphology, and calcium signaling. The major novelty in my opinion are the interaction of the two proteins, the regulation of synaptic ER by their interactions, and the methodological use of superresolution microscopy. However, these aspects would need to be developed in more depth for the study to reach its full potential. I suggest the following major revisions:*
Our response to Reviewer #3's general comments:
We very much appreciate Reviewer #3's positive comments and strong support.

Reviewer #3's major point #1. *- in Figure 5, superresolution images are shown as examples, but not employed throughout. Using superresolution to quantify the detailed morphological alterations in dendritic ER would be very interesting.*
Reviewer #3's major point #2. *- In Figure 8 as well, superresolution images and an IMARIS 3D reconstruction are shown as examples, ut not analyzed extensively. I would suggest to use superresolution throughout and employ detailed quantification of the changes they observe.*
Our response to Reviewer #3's major point #1 and #2:
Reviewer #3's first two suggestions are similar, with one referring to Figure 5 and the other to Figure 8. Consequently, we respond to them together below.

We wish to clarify that only **Figures 6C and 6D** in the original manuscript represent super-resolution images acquired using LSM980 with AiryScan2. The remaining images were acquired using a LSM700 system. We agree with Reviewer #3 that it would be very interesting to investigate morphological alterations to ER upon KLHL17 knockout in more detail. Ideally, series sectioning and 3D reconstruction of EM images would generate the most elaborate and fine-scale structures of the spine apparatus. However, this technique is not available in our labs and we were unable to establish them

within the timeframe for revision. Moreover, we do not have accessibility to STORM and STED techniques. Therefore, as an alternative approach, we combined LSM980 with Airyscan2 and expansion microscopy to acquire super-resolution images, as per our response to **Reviewer #2's major point #4**. The resolution of our LSM980 system with Airyscan2 is 90~120 nm. Since we were able to expand our cultured neurons 3.5-fold, (**New Figure 10A**), our expansion microscopy approach endowed a resolution of up to 26~35 nm.

Using this state-of-the-art microscopy system, we have now collected super-resolution images and have summarized the new data as **new Figure 10** in the revised manuscript. Our expansion protocol isotropically expands neurons, enabling observations of the entire and continuous neuronal morphology after expansion (**New Figure 10A**). Fine-scale structures of the dendritic spines, filopodia and axons were also well preserved (**New Figure 10B**). We were able to clearly monitor ER signals and their distribution within dendritic spines and along dendrites at an even better resolution (**New Figure 10**). Given that the filopodia of dendritic spines usually do not contain ER, we only analyzed spines with heads >2 μm after expansion (*i.e.*, >0.57 μm before 3.5-fold expansion). We categorized the distribution pattern of the spine apparatus into four groups, *i.e.*, cluster, sparse, neck and none. "Cluster" indicates aggregation of ER tubules at dendritic spines. "Sparse" means that ER tubules are separate from each other within dendritic spines. "Neck" represents a distribution of ER tubules along the neck of dendritic spines. "None" means no ER signal within the dendritic spine (**New Figure 10D**). Then we investigated the features of synaptic ER in wild-type neurons, Klhl17+/– neurons and wild-type neurons transfected with SYNPO-C. In WT neurons, it was easy to observe ER tubules in the dendritic spines. Similar to our conclusion in the original manuscript, Klhl17 deficiency and SYNPO-C expression reduced the percentage of ER-positive dendritic spines (**New Figure 10E**). Moreover, the cluster type of ER distribution was notably reduced in Klhl17+/– neurons and in SYNPO-C expressing neurons, and the percentages of dendritic spines lacking ER increased in both those neuronal types (**new Figure 10F**). We detected a slight increase in the sparse type of ER distribution in Klhl17+/– neurons and SYNPO-C expressing neurons (**new Figure 10F**). Thus, these super-resolution imaging analyses support that both KLHL17 and SYNPO are critical for ER clustering within dendritic spines.

Reviewer #3's major point #3.- while the interaction between the proteins is examined using biochemical methods, there are powerful imaging tools for the analysis of these interactions within individual cells and spines. I would recommend that such a method be used, to enhance novelty and impact.

Our response to Reviewer #3's major point #3:

For the revised manuscript, we have used a newly established expansion microscopy technique to analyze the distribution of KLHL17, SYNPO and ER in dendritic spines as mentioned above. Using this new system, we observed that KLHL17 and ER/SYNPO signals are either adjacent to each other or intermingled. This new data has been included as **new Figure 10C** in the revised manuscript.

We very much appreciate Reviewer #3 for her/his suggestion on super-resolution imaging analysis, which has further strengthened our conclusions and enhanced the novelty of our study.
