# Supplementary material for: Autism-related KLHL17 and SYNPO act in concert to control activity-dependent dendritic spine enlargement and the spine apparatus
Source: PLoS Biol. 2023 Aug 31;21(8):e3002274. doi: 10.1371/journal.pbio.3002274 (PMC10499226; doi:10.1371/journal.pbio.3002274)
Supplement: Attachment — Submitted filename: Our_response_to_Reviewers_20230707.docx [file pbio.3002274.s009.docx]

**Academic Editor’s comment**

The Academic Editor notes that additional justification needs to be provide for the per-spine analysis as in this case the variability appears to be on a cell-by-cell bases. If you show data per-spine, additional information needs to be included as noted by Reviewer 3. There is no justification for per-dendritic segment as these segments are part of the same cell, and appear to vary as a group depending from what cell they came. Therefore, this analysis should be re-done on a per-neuron basis.

**Our response to Academic Editor**

We thank the Academic Editor for her/his comments. Based on Reviewer 3’s suggestion, we have now used “N” to represent the number of examined neurons and “n” to indicate the number of dendritic segments or dendritic spines in all figures where applicable. All sample sizes are now clearly labeled in the second revision. Please also see our response to Reviewer #3 below.

Based on the Academic Editor’s suggestion, we have further re-analyzed dendritic spine density on a per-neuron basis and present the results of both our per-neuron and per-dendrite-based analyses in Figures 2C, 2G, 7J and 9F. The conclusion that KLHL17 and the interaction between KLHL17 and SYNPO do not influence dendritic spine density remains the same. Note that although the sample sizes of the per-neuron-based analyses are reduced by two-thirds, the variations (i.e., SEM) are similar between the per-dendrite- and per-neuron-based results because each data point is the average of three dendritic segments from the same neuron in the per-neuron analysis, resulting in reduced variation. Therefore, even though the sample size is reduced to a third, the conclusion is not altered.

We would like to take this opportunity to explain how we analyze neuronal morphology. We routinely collect neuronal images from at least two independent preparations in a blind fashion, which avoids potential personal bias. Hippocampal cultures contain different types of neurons, such as CA1, CA3 and DG. The density and size of the dendritic spines vary among these different types of neurons. Based on the morphological features of neurons, we noticed that median-sized neurons having a soma diameter ~15-18 μm and 3-8 primary dendrites are the major population in our hippocampal culture. Therefore, we selected this group of neurons for analysis. Based on the size of the soma and dendritic arbor, we speculate this type of neuron represents CA1 cells. Given that we likely analyze the same type of neurons, we thereby minimize the variation attributable to cell types. For each neuron, three clearly recognizable dendrites of comparable thickness are chosen to quantify dendritic spine density and size. Since the distance from the soma to the dendritic spine influences the density and size of dendritic spines, we specifically analyze 20-μm dendritic segments that are 20 μm apart from the soma. The spine number of those 20-μm dendritic segments represents the dendritic spine density. All dendritic spines on the selected dendritic segments are subjected to width and length analysis to avoid any potential bias. Based on all these criteria, we thus minimize in-group variation as well as personal bias, rendering our analysis both objective and reliable. Consequently, any effect of genetic variation or treatment is robustly and clearly observable. To make these points clear, we have now added the above criteria to the second revision (in the section “Immunofluorescence analysis” of the “Methods”).

Certainly, our analysis does not cover all of the different types of neurons in hippocampal cultures. Instead, it represents the results for the major neuronal population in the cultures. To dissect the effect on different types of neurons, we consider that an *in vivo* analysis using brain sections would represent a better experimental system. However, that is beyond the scope of the current study.

**Our response to Reviewers’ comments**

**Reviewer #1:**

The authors have incorporated my suggestions and improved their data analysis. I still have some questions about the calcium imaging data presented in Fig. 4: 
Fig. 4F: I assume the length of the dF/F scale bar is equal to 1 (100% fluorescence change, doubling of absolute intensity). The spine-only transient then has an amplitude of about 0.75. It is not stated whether this example is from WT or Klhl17+/- (please add this information). In Fig. 4I, however, none of the individual data points is below 1 dF/F. What is going on? Please show a representative example from the middle of the distribution. 
Also, in Fig. 4I, please always plot the amplitude in the spine (should be close to zero for dendrite-only events, right?).

**Our response to Reviewer #1:**

The example in Figure 4F is from a WT neuron. This is now labeled in the second revision of our manuscript. The height of the spine-only peak shown in Figure 4F is indeed ~0.75 dF/F. Since we set the relative amplitude of the basal level as 1 for further quantification analysis, the relative amplitude of all peaks should be greater than 1. The example of the spine-only peak in Figure 4F should be located at the position of ~1.75 in Figure 4I. Actually, we only selected the peak with a relative amplitude >1.5 for analysis, which minimized the potential noise of the recording. We apologize for not providing that information and have now included it and labeled the Y-axis as “Relative amplitude”.

We agree with Reviewer #1 that since our focus is the response at dendritic spines, the dendritic calcium dynamics of dendrite-only events are not critical. Therefore, we have removed from Figure 4I (“dendrite-only” in original Figure 4I) the data on the dendritic response for events only happening at dendrites. Doing so does indeed avoid potential confusion.

Finally, we appreciate very much Reviewer #1’s insightful comments, which have allowed us to clarify and correct some points about our calcium imaging experiments.

**Reviewer #2:**

The authors answered to questions raised by the reviewers.

**Our response to Reviewer #2:**

We very much appreciate Reviewer #2’s comments and support.

**Reviewer #3:**

The authors present a well-thought-out study with logical progression from establishing the activity-dependent function of KLHL17 in neurons to describing how its interaction with SYNPO and the ER participates in the regulation of dendritic morphology and calcium dynamics. The importance of the research is underscored by the relevance for human disease as well as the discovery of a novel function for the brain-specific protein KLHL17.  The impact of the study is improved as a result of the authors' response to previous review comments with the inclusion of additional experiments to support their conclusions. In particular, the super-resolution images reveal additional structural information which is very relevant. Some minor improvements could be made.

**Point 1:** Regarding Fig 10, the authors comment that KLHL17 signal is adjacent to SYNPO/ER, as well as intermingled together in complexes. I suggest that they quantify the percentage of single colocalization with either SYNPO or ER, as well as the triple complex.

**Our response to Reviewer #3’s comment 1:**

Although our results show a clear functional interaction between KLHL17, SYNPO and ER, we consider the interaction between KLHL17 and SYNPO to be indirect because the co-immunoprecipitation efficiency between KLHL17 and SYNPO is very low. This point was discussed in the original manuscript and the evidence was further strengthened by our expansion microscopy analysis (Please see the respective discussion in Lines 415-422 of the revised manuscript). In Figure 10C, SYNPO extensively intermingles with ER in the spine apparatus, which is consistent with previous studies showing that SYNPO acts as a marker of the spine apparatus, thus supporting the reliability of our expansion microscopy analysis. In contrast, only some KLHL17 intermingles with a proportion of SYNPO (such as in spine 2 of Figure 10C). The colocalization of SYNPO and KLHL17 revealed by expansion microscopy was very low. Therefore, we did not perform a colocalization analysis. Based on Reviewer #3’s suggestion, we have quantified colocalization of KLHL17/SYNPO, KLHL17/ER and SYNPO/ER in spines 1 and 2 shown in Figure 10C. We measured each Z-section and plotted their correlation coefficients and colocalization percentage. No matter KLHL17/SYNPO or KLHL17/ER, their correlation and colocalization were much lower than those of SYNPO/ER, further supporting the indirect interaction between KLHL17 and SYNPO/ER. To emphasize this point, we have further modified our description to “Only a very small proportion of KLHL17 signals were intermingled with ER and SYNPO (**Fig 10C, S3 Fig**).” in Lines 358-359. These new results are now included as new S3 Figure. The original S3 and S4 Figures have become S4 and S5 Figures, respectively.

**Point 2:** I agree with Reviewer #2's comment regarding the fact that for some experiments it would be more appropriate to do the statistical analysis per neuron instead of per spine, for example for the calcium imaging in Fig. 4A-E, Fig. 7A-H, Fig. 9A-D. However, I think that the per spine analysis can still be accepted provided that the authors analyze a similar number of spines for each individual neuron and that they mention the number of neurons included per condition in the figure legends.

**Our response to Reviewer #3’s comment #2:**

We thank Reviewer #3 for this comment and suggestion. In the second revised version of our manuscript, we use “N” to indicate the number of neurons and “n” to indicate the number of examined dendrites or dendritic spines. The information about sample sizes, whether neuron number or dendritic spine number, is now clearly indicated in the figures or figure legends.

We would like to emphasize that we routinely collect neuronal images from at least two independent preparations in a blind fashion. The hippocampus contains different types of neurons, including CA1, CA3 and DG. The density and size of dendritic spines vary among these different types of neurons. Based on their morphological features, we noticed that the median-sized neurons with a soma diameter of ~15-18 μm and 3-8 primary dendrites are the major population in our hippocampal cultures. Therefore, we selected this group of neurons for analysis. For each neuron, we choose three clearly recognizable dendrites of comparable thicknesses to quantify dendritic spine density and size. Given that the distance from the soma to the dendritic spine influences the density and size of dendritic spines, we further specifically analyze 20-μm dendritic segments that are 20 μm apart from the soma. The spine number of those 20-μm dendritic segments then represents the dendritic spine density. All dendritic spines on the selected dendritic segments are subjected to width and length analysis. In doing so, we minimize in-group variation as well as personal bias. This approach also renders our analysis both objective and reliable. To make this point clear, we have now added the above criteria for analysis into the second revision of our manuscript (in the section “Immunofluorescence analysis” of the “Methods”).

Point 3: The legend for S1 figure does not match the panels. Please correct.

Point 4: Page 3 "actifilin" spelling

**Our response to Reviewer #3’s comments #3 and #4:**

The legend for Figure S1 and the misspelling of “actifilin” on Page 3 have now been corrected. We very much thank Reviewer #3 for these points.
